# Commentary on CDC data showing an increased risk for pediatric diabetes with COVID‐19 infection

**DOI:** 10.1111/1753-0407.13311

**Published:** 2022-09-08

**Authors:** Jasmine Gujral, William Tamborlane, Laura Nally

**Affiliations:** ^1^ Division of Pediatric Endocrinology and Diabetes, Department of Pediatrics Yale School of Medicine New Haven Connecticut USA

## Abstract

**Highlights**

- We question certain aspects of the recent Centers for Disease Control and Prevention (CDC) Morbidity and Mortality Weekly Report (January 2022) showing a significant increase in the incidence of diabetes in children after COVID‐19 infection.
- We are concerned at the source of data being limited to commercial health insurances and lack of factoring race, ethnicity, social determinants of health, body mass index, type of diabetes, and viral variants in the interpretation of these data.

It is well known that multiple viruses (cytomegalovirus, coxsackievirus, enterovirus) are associated with triggering development of type 1 diabetes in genetically susceptible individuals. In January 2022, the CDC, in their Morbidity and Mortality Weekly Report, published a study suggesting that youth under 18 years old had a higher rate of developing diabetes more than 30 days after COVID‐19 infection.^1^ In this study, two large commercial insurance claims databases (IQVIA and HealthVerity) were utilized for retrospective review to calculate the incidence of new onset diabetes more than 30 days after an acute infection with SARS‐CoV‐2. For IQVIA, data were collected from March 2020 to February 2021, and the incidence of new onset diabetes was compared among patients diagnosed with COVID‐19, matched by age and sex with patients who were not diagnosed with COVID‐19 during the pandemic or who received a non–COVID‐19 acute respiratory infection (ARI) diagnosis prior to the pandemic (hazard ratio [HR] = 2.66, 95% CI = 1.98‐3.56). For HealthVerity, data were collected from March 2020 to June 2021, and the incidence of diabetes was calculated in health care encounters possibly related to COVID‐19 (HR = 1.31, 95% CI = 1.20‐1.44).

Significantly higher rates of diabetic ketoacidosis (DKA) were noted in patients with a previous COVID‐19 diagnosis who went on to develop diabetes, 40.2% (HealthVerity) and 48.5% (IQVIA), compared to DKA reported in patients with new onset diabetes who did not have a recent COVID‐19 diagnosis (IQVIA: non‐COVID 13.6%; ARI 22.0%; non‐ARI 27.5%; HealthVerity: 29.7%).

The report's strengths are that it utilizes large sample sizes (1.6 million and 0.8 million for each database, respectively) and captures data in the USA since the advent of the pandemic for at least 12 months. Having age‐ and sex‐matched control groups adds to the strengths of the study. It also corroborates with prior US data showing an increased risk for diabetes in the adult population with COVID‐19 and is consistent with studies from Europe that similarly report an increased incidence of pediatric diabetes associated with COVID‐19 infection. Further, the incidence of diabetes in the non‐COVID cohort in the IQVIA database was 32.6/100000 children, consistent with data from the SEARCH study, which reported incidence of diabetes in youth at 36.1/100000 children.^2^ The report is also consistent with previous data citing increased frequency and severity of DKA at time of diagnosis of both type 1 and type 2 diabetes during the pandemic, thought to be multifactorial and related to delayed care seeking, worsening of structural health disparities, and to a certain extent, the diabetogenic effects of COVID‐19.^3^, ^4^, ^5^

The authors appropriately list several limitations that are important to consider. Significant details regarding glycosylated hemoglobin levels, presence of autoantibodies, and characterization of diabetes into type 1 or type 2 were not presented. Patients were only followed for limited periods of time and longer‐term follow up would be required to assess whether diabetes was transient or persistent. It is well established that the risk for both types of diabetes varies significantly between different racial and ethnic groups and that COVID‐19 has disproportionately affected disadvantaged groups.^6^ Unfortunately, important details addressing race, ethnicity, presence of comorbid conditions, socioeconomic factors, and body mass index (BMI) were not reported. Several studies have shown an alarming rise in childhood obesity and BMI since the beginning of the pandemic, attributable to various factors such as limited physical activity, virtual schooling, disrupted routines, and food insecurity.^7^ Many authors suggest an increase in type 2 diabetes in children from accelerated weight gain. A factor that could potentially misrepresent the true incidence of new onset diabetes cases associated with COVID‐19 infection is that in the beginning of the pandemic in the USA (March 2020), testing was not universally available or standardized and asymptomatic children were not being tested.

Additionally, the data do not represent uninsured children (5%) or those on Medicaid (29%), who encompass over one‐third of the pediatric population in the USA, a substantial portion that may be disproportionately affected by COVID‐19 and type 2 diabetes. Thus, the true incidence of diabetes may be much higher, and risk of developing type 1 vs type 2 diabetes is likely quite different and considerably more complex than what we can glean from the data presented.

The timing of presentation for diagnosis of diabetes should also be considered. Because children with prediabetes and diabetes are often asymptomatic, diabetes may have been incidentally diagnosed when presenting to the clinician with COVID‐19 symptoms. Thus, the acute infection may have brought children to medical attention sooner, allowing for the diagnosis of diabetes to be made at presentation.

Also, this report is only applicable to the COVID‐19 viral variants present from March 2020 to June 2021 and does not represent the omicron surge that has been sweeping our country and has affected larger numbers of children than any prior wave of the pandemic.

Lastly, while the conclusions in the article highlight vaccination as a strategy to prevent new onset diabetes after COVID‐19 infection, this study did not evaluate how vaccination status affected diagnoses of new onset diabetes in the pediatric population. While vaccination is important to help prevention of well‐documented sequelae, to our knowledge, there are no data to support that vaccination will prevent diabetes, and this takeaway message should be interpreted with caution.

In conclusion, this observational study presents preliminary data of an association between new onset diabetes and recent COVID‐19 infection; it does not establish causation. Further prospective studies are required to confirm these findings.

## FUNDING INFORMATION

None.

## DISCLOSURES

The authors have no conflicts of interest to disclose.
